# Bacteriophage-Insensitive Mutants of Antimicrobial-Resistant *Salmonella Enterica* are Altered in their Tetracycline Resistance and Virulence in Caco-2 Intestinal Cells

**DOI:** 10.3390/ijms21051883

**Published:** 2020-03-10

**Authors:** Karen Fong, Kaiwen Mu, Jean-Guillaume Rheault, Roger C. Levesque, David D. Kitts, Pascal Delaquis, Lawrence Goodridge, Siyun Wang

**Affiliations:** 1 Food, Nutrition and Health, The University of British Columbia, Vancouver, BC V6T 1Z4, Canada; karen.fong@ubc.ca (K.F.); muk@mail.ubc.ca (K.M.); david.kitts@ubc.ca (D.D.K.); 2 Institut de biologie intégrative et des systèmes (IBIS), Université Laval, Quebec City, QC G1V 0A6, Canada; jean-guillaume.emond-rheault.1@ulaval.ca (J.-G.R.); rclevesq@ibis.ulaval.ca (R.C.L.); 3 Agriculture and Agri-Food Canada, Summerland, BC V0H 1Z0, Canada; pascal.delaquis@canada.ca; 4 Food Science Department, University of Guelph, Guelph, ON N1G 2W1, Canada; goodridl@uoguelph.ca

**Keywords:** phage resistance, antibiotic resistance, virulence, *Salmonella*

## Abstract

Bacteriophages have shown promise as therapeutic alternatives to antibiotics for the control of infectious bacteria, including the human pathogen *Salmonella.* However, the development of effective phage-based applications requires the elucidation of key interactions between phages and target hosts, particularly since host resistance to phage is inevitable. Little is known about the alteration of host phenotypes following the development of resistance to phage. The aim of this study is to evaluate the antibiotic susceptibility and virulence of a *Salmonella* isolate following the development of resistance to bacteriophage SI1. We observed enhanced susceptibility to tetracycline and decreased invasion capacity in a differentiated Caco-2 intestinal cell line. Whole genome sequence analysis revealed an array of mutations, most notably, truncations in *vgrG1_2*, a core gene involved in Type VI secretion and mutations in the lipopolysaccharide, thereby indicating the plausible attachment site of phage SI1. These findings shed light on understanding the underlying mechanism for phage immunity within the host. Importantly, we reveal an associated genetic cost to the bacterial host with developing resistance to phages. Taken together, these results will aid in advancing strategies to delay or eliminate the development of host resistance when designing informed phage-based antimicrobials.

## 1. Introduction

The foodborne pathogen non-typhoidal *Salmonella* causes 93 million enteric infections, resulting in more than 150,000 deaths and 4,847,000 disability-adjusted life years lost worldwide per annum [1,2]. Salmonellosis is generally self-limiting in developed countries, although chronic complications may arise, including reactive arthritis and chronic gastroenteritis [3]. The ability of this pathogen to colonize diverse niches has led to its persistence and survival in a range of foods that may serve as vehicles of transmission, including poultry, eggs, meat, dairy products, fresh produce, and a variety of ready-to-eat products [4,5]. Given the frequency of infections and the multiplicity of potential vehicles of transmission, the rise of antimicrobial resistance (AMR) in *Salmonella* is of particular concern. [6,7]. Since 2017, AMR *Salmonella* has been ranked among the top ten pathogens of high priority by the World Health Organization and is considered an imminent threat to public health [6].

High research and development costs, poor time-to-market performance, and lack of profitability have long hindered private sector investment in novel antibiotic discovery [8,9]. Consequently, interest in the use of bacteriophages (phages) as alternative therapeutic agents for the treatment of infections caused by AMR bacteria has increased in recent years [7]. Successful use of phages for the control of antimicrobial-resistant bacteria has been reported in several contexts, including human medicine [7,10] and the food industry [11]. Unfortunately, the emergence of bacteriophage-insensitive mutants (BIMs) can occur upon repeated exposure of bacterial hosts to specific phage strains [12,13]. BIMs may acquire phage resistance through several means, such as restriction-modification, abortive infection, CRISPR/Cas9 mechanisms, and commonly, through mutations in phage receptor sites, thereby preventing phage attachment [14]. Additionally, it is known that developing resistance to phage may come with an associated environmental fitness cost to the host, where the evolution of an advantageous trait simultaneously compromises performance in another trait [12,13]. Slower growth rates [15,16], decreased virulence [12], and diminished resistance to various antimicrobials [17] have been reported in BIMs of various bacterial species. Fitness costs have been shown to vary across genera and species of bacteria [18], although only nominal work has been performed with foodborne pathogens such as *Salmonella*. We hypothesize here that BIMs of *Salmonella* may exhibit increased susceptibility to antibiotics and decreased virulence potential.

## 2. Results and Discussion

### 2.1. General Characterization of S. Agona BIMs

To isolate putative BIMs of *S*. Agona, the screening assay was performed 200 times. Overall, the rate of resistance development was 2.5% when SI1 was used as the infecting phage, although a transient, unstable resistance phenomenon that disappeared upon further propagation was often observed. Five BIMs of *S*. Agona FSL S5-517 were isolated and designated ∆87, ∆95, ∆96, ∆99, and ∆102. In contrast with previous reports [15,16], there were no apparent differences in the growth rates of the BIMs and the parental strain (Figure S1). A range of mutations (including single nucleotide polymorphisms (SNPs) and insertion/deletions (indels)) were observed in all BIMs, although the number of mutations and mutated genes varied (Tables S1 to S5 inclusive). Interestingly, some genes and loci were highly mutable across all BIMs (e.g., *ccm* family genes, *vgrG, solA, sufD*), suggesting common mechanisms of cellular modification with phage resistance.

### 2.2. Lipopolysaccharide Mutations in BIMs

Two mutants, ∆95 and ∆96, were found to have several identical mutations in *rfaL* and *rfaY*, respectively, encoding an O-antigen ligase and lipopolysaccharide (LPS) core heptose II kinase, proteins involved in LPS synthesis. Variants in these loci were comprised of SNPs and insertions of varying lengths, which induced non-synonymous, synonymous, and frameshift mutations (Table 1). As no other mutations were observed in surface-associated molecules, it is likely the LPS is the receptor site of phage SI1. *Salmonella* type phage Felix-O1, capable of infecting 99% of Salmonellae, has also been shown to utilize the bacterial LPS for adsorption [19], and mutations in loci responsible for LPS synthesis and structure have been observed in BIMs of numerous bacteria, including *Salmonella* [19], *Yersinia pestis* [12] and *E. coli* [20]. Mutations within *rfaL* disrupt O-antigen biosynthesis and have been reported to induce phage resistance in *S*. Typhimurium [21,22] and *E. coli* O157:H7 [21]. Alternatively, deletions identified within *rfaJ*, *rfaI*, *rfaG*, *rfaF*, and *rfaC* disrupted synthesis of the LPS outer core and induced host resistance to *Salmonella* phage SSU5 [19]. Given the available literature, it is unclear if mutations in *rfaY* would induce a significant change in the LPS core and if this would further result in phage resistance. Therefore, the current data suggest that the O-antigen of the LPS is the site of attachment by phage SI1, although the possibility of outer core specificity cannot be definitively ruled out.

Mutations in genes involved in LPS synthesis were not identified in ∆87, ∆99, nor ∆102, although they demonstrated resistance to phage SI1 (Table 1). It is hypothesized that these strains possess adaptive immunity mechanisms that obscure the correlative relationship between LPS mutations and phage resistance, for instance, CRISPR and/or other adaptive strategies (e.g., restriction-modification, superinfection exclusion). Therefore, we used CRISPRFinder [23] to identify CRISPR elements in the genomes of the wild-type (WT) and derivative strains. Although there were 26 CRISPRs identified (Table S6), the spacer sequences did not possess homology to any known phages, including SI1. It is possible that other innate immunity mechanisms, including restriction-modification, superinfection exclusion, and abortive infection, could play a role in the phage resistance of these strains [14]. Future work should aim to identify and functionally characterize known and novel gene cassettes contributing to this phenotype.

### 2.3. Antibiotic Sensitivity of BIMs

Compared to the WT strain, BIMs were not altered in relative sensitivities to sulfamethoxazole/trimethoprim (SXT) nor sulfisoxazole (SUF), exhibiting MIC values of 180/3500 and 2048 μg/mL, respectively (Table 2). According to the designated breakpoint values of the Canadian Integrated Program for Antimicrobial Resistance Surveillance (CIPARS), these MIC values indicate resistance to SXT and SUF [24].

Interestingly, ∆96 showed enhanced sensitivity to tetracycline (TET), exhibiting a MIC_TET_ of 76.8 μg/mL compared to an MIC_TET_ of 128 μg/mL for the other isolates. Despite a 60% reduction in MIC, ∆96 is still categorized as TET-resistant according to CIPARS (2016). However, reasons for the increased sensitivity of ∆96 to TET are not immediately apparent. 

Enhanced antibiotic sensitivity may occur in phage-resistant bacteria if a cellular target serves as the recognition site for both the antibiotic and the phage [17]. In a targeted approach, phages infecting multi-drug resistant *Pseudomonas aeruginosa* were isolated that utilized multidrug efflux pumps as receptors [17]. Phage-resistant mutants of *P. aeruginosa* that possessed mutations in these regions subsequently exhibited enhanced susceptibility to several classes of antibiotics, including ceftazidime, ciprofloxacin, tetracycline, and erythromycin, providing evidence of a genetic trade-off as a result of phage infection [17,25].

As previously noted, the presence of mutations in genes associated with LPS synthesis in the BIMs suggested that the receptor site of phage SI1 is associated with this locus (Table 1). As TET specifically binds to regions of the 16S rRNA molecule [26], the direct development of sensitivity is likely not associated with LPS synthesis nor modification. However, it is also known that there are cellular costs associated with phage immunity [14,18,27,28].

Of the four genes encoding proteins involved in TET resistance (i.e., resistance proteins class B (1), C (2), and TET regulator class A (1)) identified in *S.* Agona FSL S5-517, no mutations were observed as a result of developing phage resistance (Tables S1 to S5 inclusive). It is possible that alterations in transcriptional activity may account for the observed phenotype. For instance, significant (*p* < 0.05) downregulation of three antibiotic resistance genes were observed by global transcriptomic analysis of multi-drug-resistant *Acinetobacter baumanii* infected with phage ∂Abp1 [25]. The enhanced expression of efflux pumps in both phage-resistant *Campylobacter jejeuni* and P. *aeruginosa* has also been documented [29,30], leading to speculation that efflux pumps and other AMR proteins may have dual roles in phage resistance. As lytic phages are currently being considered for control of antimicrobial resistant bacteria, further work into understanding the influence of phage resistance on antibiotic resistance genes (and particularly, those of AMR *Salmonella*) is clearly warranted.

### 2.4. Adhesion and Invasion Assays

Adherence represents an important initial step in epithelial cell invasion [31]. Rates of adherence of the BIMs were not significantly (*p* > 0.05) different than those of the parental strain (Table 3). Concordantly, no observed mutations were present in structures involved in adherence (e.g., pili, flagella; Tables S1 to S5 inclusive). There were, however, a number of mutations observed in various secretion systems (e.g., Type IV and Type VI secretion systems) across the different BIMs. Nevertheless, it appears that the adhesion proteins involved in these secretion systems were not affected (Tables S1 to S5 inclusive). To the best of our knowledge, there are currently no published reports regarding the adhesion properties of phage-resistant *Salmonella*; therefore, these results shed light on phenomena that may underlie phage resistance. It is interesting to speculate, however, that cell adhesion may be affected if adhesion- associated structures (e.g., pili, flagella) were the site of phage adsorption.

**Figure 1:**
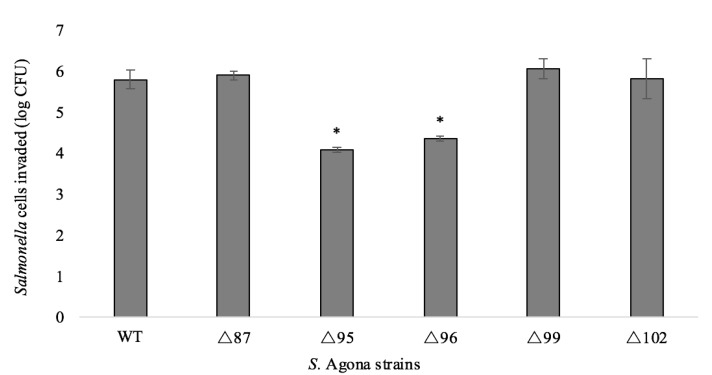
Caco-2 cell invasion capacity by the *S.* Agona strains. Error bars indicate the standard deviations of the means of three biological replicates. Means of the BIMs were compared to that of the WT. Asterisks located above mean values indicate significance below α = 0.05 (one-way ANOVA).

BIMs of *S.* Agona displayed varied invasion efficacies (Figure 1). Compared to the WT strain, the invasion capacities of ∆87, ∆99, and ∆102 were not significantly affected (*p* > 0.05). However, the number of invaded cells of ∆95 and ∆96 showed a decrease of 1.71 ± 0.29 and 1.45 ± 0.31 log CFU, respectively. Attenuated virulence in phage-resistant bacteria has been noted previously. For example, loss of the O-polysaccharide on the LPS of *S.* Enteritidis conferred resistance to phage infection and resistant mutants were avirulent when tested with the *Caenorhabditis elegans* virulence model [32]. Moreover, it is known that *S*. Typhimurium requires an intact O-polysaccharide to trigger programmed cell death in *C. elegans* [33], and loss of this constituent was correlated with loss of virulence. Similarly, the outer core of the LPS is required for entry of *S*. Typhi into epithelial cells [34]. Although we also observed mutations in the LPS biosynthesis genes (Table 1), our results are not directly comparable because we did not use a systemic model of infection. Mutations in other cellular structures contributing to phage adsorption (e.g., flagella, outer membrane proteins) have also been associated with attenuated virulence [35,36,37,38]. Furthermore, it is known that the maintenance of innate and adaptive bacterial immunity (e.g., restriction-modification, CRISPR-Cas systems) has associated cellular costs [27,28].

**Figure 2:**
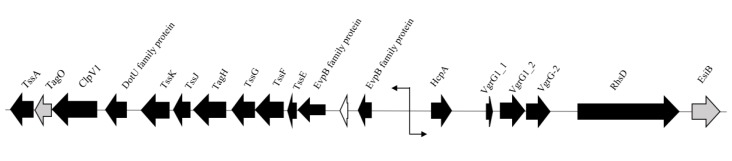
Schematic representation of the T6SS gene cluster in *S.* Agona FSL S5-517. Arrows indicate direction of transcription. Black arrows indicate gene products involved in the T6SS. Grey arrows represent gene products of unknown function but believed to play a role in the T6SS. White arrow indicates a hypothetical protein.

Variant analysis revealed several mutations in components involved in the Type VI secretion system (T6SS) of SPI-19 that plays a role in intestinal infection (Table 1) [39,40,41]. Homology between the contractile components of phages and T6SS of several *Salmonella* serovars that deliver cytotoxic effector proteins via spikes that enter the host cell was recently reported [42,43,44]. Many core structural components, however, have not been extensively characterized on a functional level [45]. Genes encoding Hcp (hemolysin coregulated protein) and three non-identical copies of VgrG (VgrG1_1, VgrG1_2, and VgrG-2) were identified in *S*. Agona FSL S5-517, all of which are associated with the tube and needle-like apparatus of the T6SS [46,47]. Given the dual functionality of VgrG in T6SS structure and cytotoxicity [47,48], it is unclear if these genes are individually involved in tip structure, host cytotoxicity, or both. Downstream of the core genes is an rhs element that likely encodes a secretion apparatus [48]. Upstream, the *tss* locus was identified, encoding for structural proteins involved in the formation of the T6SS baseplate [45]. Interestingly, the switch in the direction of transcription indicates that different sections of the T6SS apparatus are transcribed and assembled separately (Figure 2).

VgrG proteins, in particular, are believed to possess dual functionality as both a cell-puncturing device [47] and a secreted effector protein, resulting in intracellular toxicity, most notably through actin modification [48]. It has been shown that the T6SS of *S*. Gallinarum is required for survival in the infected macrophages of chicks [39]. Additionally, vgrG deletion mutants of *A. baumanii* were compromised in adhesion, growth rate, and invasion [41]. Interestingly, these mutants also showed enhanced susceptibility to chloramphenicol, a novel finding that demonstrates the dual roles of T6S in both virulence and AMR [41].

Mutation hotspots, regions where mutations clustered in close proximity, were observed in all BIMs regardless of invasion efficacy, indicating that *vgrG* and/or regions therein may be highly mutable in conditions where phage is present (Table 1). In all BIMs, a six-bp insertion induced a frameshift at nucleotide position 792 of *vgrG1_2*, indicating the selective pressure to accumulate indels in multiples of three in an attempt to preserve the reading frame [49,50]. Three downstream polymorphisms also resulted in the conversion of three amino acids (Table 1). Interestingly, these mutations did not affect the invasion capacities of ∆87, ∆99, nor ∆102, indicating that these variant sites may preserve the protein structure and/or deleterious effects were minimized due to the indel’s close proximity to the 3′ end of the gene (951 bp in length) [43].

Strains ∆95 and ∆96 were shown to be significantly less invasive (*p* < 0.05) than the WT strain (Figure 1) and we observed a higher number of mutations in the *vgrG* genes than the other BIMs (Table 1). Notably, a G to T substitution at nucleotide position 823 induced the appearance of a stop codon in both BIMs, resulting in a truncated protein and potentially negating any neutral effects of upstream mutations we observed in all BIMs. As mutations were only observed in *vgrG1_2*, it is possible that *vgrG1_1* may partially compensate for the loss of invasion efficacy, particularly since the complete loss of virulence was not observed in the present study. However, the extent of this compensation is unknown since the proteins do not share an identity and may possess different functions. To our knowledge, this is the first report of mutations in the T6SS as a result of phage resistance in non-typhoidal *Salmonella.*

Differential patterns of gene expression following phage resistance have also been investigated in several studies, although there is a paucity of literature regarding the attenuation of virulence in phage-resistant, non-typhoidal *Salmonella*. In a global transcriptome analysis of phage-resistant *A. baumanii*, the expression of *vgrG* was downregulated by two-fold and a subset of seven other genes involved in Type II, V, and VI secretion were also significantly downregulated [25]. Upregulation of virulence factors as a result of phage resistance has been reported elsewhere [51,52].

Phage-resistant variants of *P. aeruginosa* PAO1 displayed an upregulation of up to 108.4-fold in key genes involved in Type II, III, and VI secretion, which was confirmed with the decreased viability of infected mammalian cells. Given the diverse phenotypes associated with phage resistance, the assessment of virulence potential in *Salmonella* BIMs is clearly warranted, especially when considering human safety in biocontrol applications.

## 3. Materials and Methods

### 3.1. Bacterial Maintenance & Growth Conditions

An *S*. Agona strain (FSL S5-517, human isolate) resistant to SUF, SXT, and TET was used to examine the effect of phage resistance on AMR. Stocks were maintained at −80 °C in brain–heart infusion broth (BD/Difco, East Rutherford, NJ, USA) supplemented with 20% glycerol. Working stocks were prepared on tryptic soy agar (TSA; BD/Difco, East Rutherford, NJ, USA) and maintained at 4 °C for a maximum of one month. Prior to experiments, fresh overnight cultures were prepared by inoculating a single colony into 5 mL tryptic soy broth (TSB; BD/Difco, East Rutherford, NJ, USA). Cultures were incubated for 20 h (stationary phase) at 37 °C with agitation at 170 rpm.

### 3.2. Isolation of BIMs of S. Agona FSL S5-517

Phage SI1, an obligate lytic phage, was previously isolated and characterized in our laboratory as previously described [5]. It was selected for this study due to its strong infectivity against *S.* Agona FSL S5-517 [5]. Pure phage lysates were stored at 4 °C in SM buffer until further analysis [5].

Putative BIMs were isolated according to methods described by Pereira et al. [53]. Briefly, 5 μL of a phage SI1 suspension (~10^9^ PFU/mL) were spotted onto bacterial lawns consisting of 300 μL of stationary phase cultures of *S.* Agona mixed with 4 mL 0.7% TSA overlaid onto 1.5% TSA in Petri plates. The plates were incubated at 37 °C for 48 h to allow development of colonies (i.e., putative BIMs) within lysis zones. Individual colonies were picked and transferred to 5 mL TSB, grown overnight at 37 °C for 20 h and were applied to TSA. Bacterial lawns were simultaneously prepared using the overnight cultures as described above, and 5 μL of phage SI1 (10^9^ PFU/mL) were spotted onto the lawns to confirm resistance. The procedure was repeated five times to avoid selection of isolates with transient phage resistance phenotypes [19]. Stock solutions of confirmed BIMs were prepared in TSB supplemented with 20% glycerol and stored at −80°C for further analyses.

### 3.3. Minimum-Inhibitory Concentration Assays

Minimum inhibitory concentrations (MICs) of SUF, SXT, and TET against the BIMs of *S.* Agona and the phage-sensitive, WT parental strain were measured using a broth microdilution method [54]. Prior to the MIC assay, resistance of the BIMs to phage SI1 was confirmed by spotting five μL of phage SI1 (10^9^ PFU/mL) onto a bacterial lawn of *S.* Agona, followed by incubation for 18 h at 37 °C.

All cultures were grown in triplicate by incubation at 37 °C in 10 mL TSB for 20 h. Cells were harvested by spinning one ml aliquots at 5000× *g* for ten min, washed three times in phosphate buffered saline (PBS; Amresco, Solon, OH, USA) and resuspended in cation-adjusted Mueller–Hinton broth (MHB; Amresco). Aliquots were then transferred in duplicate to 96-well plates containing twofold dilutions of SUF, SXT, and TET, and successively diluted in cation- adjusted MHB (Amresco) to a final concentration of 5 × 10^4^ CFU/mL. Plates were subsequently incubated at 37 °C for at least 16 h. Growth in the wells, as indicated by turbidity, indicated resistance to the antibiotic. Controls using antibiotic-sensitive *Salmonella* strains were also included. The lowest concentration of each antibiotic which prevented growth of bacteria was deemed the MIC.

### 3.4. Caco-2 Cell Maintenance and Differentiation

Caco-2 cells (human colon enterocyte-like; HTB-37, American Type Culture Collection, Manassas, VA, USA) were maintained in Dulbecco minimum essential medium (DMEM; Sigma, St. Louis, MO, USA) supplemented with 5% fetal bovine serum (Invitrogen, Burlington, ON, Canada), 100 U/mL penicillin and 100 μg/mL of streptomycin (Sigma) at 37 °C in 5% CO_2_. Cells were sub-cultured weekly using a 1:10 split ratio with medium changes made every two to three days. The passage number used was 20–40.

For cell differentiation, a 24-well tissue culture plate (1.93 cm^2^) was seeded with Caco-2 cells at a density of approximately 5 × 10^5^ cells/well in DMEM. Cells were allowed to differentiate for 21 days in 5% CO_2_ with the culture medium changed every two days.

### 3.5. Adhesion & Gentamicin Protection Assays

*S*. Agona isolates were grown in triplicate in 5 mL TSB at 37 °C under agitation for 20 h. Cells were harvested by centrifugation at 5000× *g* for 10 min and washed three times with PBS, followed by final resuspension in DMEM without antibiotics. Prior to performance of the gentamicin protection assay, resistance of the BIMs to phage SI1 was confirmed by spotting 5 μL of phage SI1 (10^9^ PFU/mL) onto a bacterial lawn of *S*. Agona, followed by incubation for 18 h at 37 °C.

Caco-2 cells were replenished with DMEM 24 h prior to analysis in order to ensure the viability of invading bacteria. Briefly, seeded cells were washed with PBS and infected with *S.* Agona at a final concentration of 5 × 10^6^ CFU/well (multiplicity of infection = 10) in duplicate. Infected Caco-2 cells were then incubated at 5% CO_2_ for 1 h to allow for adhesion and an additional 2 h to allow for invasion [55,56]. The plates were then aspirated and washed three times with 1X PBS to eliminate non-adherent *Salmonella*.

To test for adherence, 500 μL of 0.1% Triton X-100 (Amresco) was added to each well to lyse the Caco-2 cell monolayers and gently mixed by pipetting. Then, 100 μL of the cell suspensions were serially diluted in PBS and applied to the surface of TSA in duplicate. Plates were incubated at 37 °C prior to counting colonies.

In a separate assay to test for invasion, 100 μg/mL of gentamicin (Amresco) was added to each well to eliminate adherent, non-invaded bacterial cells. Plates were incubated at 5% CO_2_ at 37 °C for 2 h. Wells were then washed twice with PBS. Subsequently, 500 μL of 0.1% Triton X-100 was added to lyse the Caco-2 cells and gently mixed to release intracellular *Salmonella*. The invading bacteria were immediately serially diluted in PBS and applied to the surface of TSA in duplicate. Plates were incubated at 37 °C and colonies were counted after 18 ± 2 h of incubation.

### 3.6. DNA Preparation, Sequencing and Variant Calling

Genomic DNA was extracted from overnight lysogeny broth cultures (Amresco) at 37 °C using the E-Z 96 Tissue DNA Kit (Omega Biotek, Norcross, GA, USA), according to the manufacturer’s instructions. Subsequently, 500 ng of genomic DNA was mechanically fragmented for 40 s by Covaris M220 (Covaris, Woburn, MA, USA) using default settings. Libraries were synthesized using the NEBNext Ultra II DNA library prep kit for Illumina (New England Biolabs, Ipswich, MA, USA) according to manufacturer’s instructions and were sequenced to obtain 30× of coverage in an fdfIllumina MiSeq 300-bp paired-end run at the Plateforme d’Analyses Génomiques of the Institut de Biologie Intégrative et des Systèmes (Laval University, Quebec, QC, Canada). Each genome was assembled de novo with the A5 pipeline version A5-miseq 20140521 [57]. Contigs were mapped to a reference genome, *S*. Agona SL483 (Accession: PRJNA20063), using Mauve version 2.4.0 [58]. Sequence data was then uploaded to the Galaxy platform, where the public server at usegalaxy.org was used for data analysis [59]. Adapters were trimmed using the Trimmomatic tool with default parameters [60]. Prokka was used for annotation of the reference genome [61] Sequencing reads of the BIMs were aligned to the WT reference genome using Bowtie2 [62], followed by local re-alignment using Realigner Target Creator [63]. Variants (indels, SNPs, MNPs) were called using FreeBayes [64]. High-quality variants were selected if they possessed a minimum mapping coverage of 8 and a minimum quality score of 20 (90%) [65].

To elucidate the presence of CRISPR-Cas mechanisms in the BIMs, CRISPRFinder was used to identify direct repeats and spacer regions [23]. The NCBI BLASTn algorithm was used to align spacer regions to the genome of phage SI1.

### 3.7. Statistical Analysis

Invasion efficacy measured by the gentamicin protection assay was calculated from the mean number of cells invaded (in CFU) for three biological replicates. Means obtained with the BIMs were compared to those of the WT strain using a one-way ANOVA. Statistical analyses were performed using JMP version 11.1.1 (SAS Institute, Inc., Cary, NC, United States). A *p*-value of ≤ 0.05 was considered statistically significant.

## 4. Conclusions

In considering employing phages for biocontrol, it is of critical importance to assess the interactions that occur between the phage and the host, particularly since an interaction may signify the success of an application. Upon phenotype assessment of spontaneous BIMs of AMR *S*. Agona, we observed enhanced susceptibility to TET and decreased invasion efficacy in a differentiated Caco-2 intestinal cell model. These phenotypes were correlated with mutations in the host LPS, indicating the site of phage attachment, and a plethora of mutations (most notably, truncations in ∆95 and ∆96) in *vgrG1_2*, a core gene of the T6SS. It is important to emphasize that this association should be experimentally confirmed in future studies using targeted genetic approaches (i.e., through functional characterization). Additionally, in vivo assays using systemic models of infection will further elucidate the virulence potential of such BIMs. Although mutations were not observed in host TET resistance genes, it can be speculated from this study that downregulation of AMR genes following phage resistance may account for the phenotype, as has been observed by others [25]. If this is the case, variant analysis would not reveal the association; however, it is expected that global transcriptomic analysis would elucidate these correlations.

Together, these results indicate that AMR and virulence phenotypes were altered in phage-resistant *S.* Agona, and highlighted phage-induced genetic tradeoffs in *Salmonella*. These data shed light on the fate of the host following phage exposure, which is pivotal for research on the composition of phage cocktails for biocontrol applications in food, animal agriculture, or human medicine. 

## Figures and Tables

**Table 1:** Mutations in genes involved in lipopolysaccharide synthesis and type VI secretion system of the *S*. Agona bacteriophage-insensitive mutants (BIMs). Mutations presented possess a minimum quality score of 20 and a minimum mapping coverage of 8.

Affected Gene	Putative Function	Strand	nt Position ^a^	Strain	Mutation	Sequence ^b^	Impact on Polypeptide Synthesis
*rfaL*	O-antigen ligase	+	425	WT	3 bp insertion	G------A	Insertion
				Δ95		GGATA	
				Δ96		GGATA	
		+	429	WT	Substitution	G	None
				Δ95		G>A	
				Δ96		G>A	
		+	442	WT	4 bp insertion	T-------A	Frameshift
				Δ95		TGGGA	
				Δ96		TGGGA	
*rfaY*	Lipopolysaccharide core heptose (II) kinase	-	514	WT	23 bp insertion	A---------------------------------------------- A	Frameshift
				Δ95		AGCGAAGCCCTAAACTTGTTAAAAA	
				Δ96		AGCGAAGCCCTAAACTTGTTAAAAA	
		-	538	WT	Substitution	T	Asn --> Lys
				Δ95		T>G	
				Δ96		T>G	
*vgrG1_2*	Actin cross-linking toxin, structural tip protein, involved in type VI secretion	+	792	WT	6 bp insertion	T-------------	Frameshift
				Δ87		TCAAGGA	
				Δ95		TCAAGGA	
				Δ96		TCAAGGA	
				Δ99		TCAAGGA	
				Δ102		TCAAGGA	
		+	798	WT	Multi-nucleotide substitution	ATTTT	Leu --> Phe Phe --> Gly Tyr --> His
				Δ87		ATTT>CGGCC	
				Δ95		ATTT>CGGCC	
				Δ96		ATTT>CGGCC	
				Δ99		ATTT>CGGCC	
				Δ102		ATTT>CGGCC	
		+	810	WT	4 bp insertion	A---------T	Frameshift
				Δ95		AGAACT	
				Δ96		AGAACT	
		+	816	WT	2 bp insertion	T----C	Frameshift
				Δ95		TCAC	
				Δ96		TCAC	
		+	820	WT	Substitution	G	Gly --> Arg
				Δ95		G>C	
				Δ96		G>C	
		+	823	WT	Substitution	G	Gly --> Stop codon
				Δ95		G>T	
				Δ96		G>T	
		+	825	WT	Substitution	A	None
				Δ95		A>G	
				Δ96		A>G	
		+	828	WT	3 bp deletion	AGGA	Deletion
				Δ95		A------	
				Δ96		A------	
		+	831	WT	Substitution	C	Asp --> Glu
				Δ95		C>G	
				Δ96		C>G	
		+	836	WT	Substitution	C	Ala --> Glu
				Δ95		C>A	
				Δ96		C>A	

^a^ relative to wild-type strain. nt: nucleotide. ^b^ presented in 5′ to 3′ direction. In substitutions, alternate sequences are preceded by the original sequence and separated with a “>” symbol.

**Table 2:** Minimum inhibitory concentrations of the *S. Agona* strains. Minimum inhibitory concentrations (MICs) were determined by conducting each test in triplicate.

Strain	MIC (µg/mL)		
	SXT ^a^	TET	SUF
Wild type	180/3500	128	2048
∆87	180/3500	128	2048
∆95	180/3500	128	2048
∆96	180/3500	76.8	2048
∆99	180/3500	128	2048
∆102	180/3500	128	2048

^a^ Values refer to the MICs of sulfamethoxazole and trimethoprim, respectively.

**Table 3:** Caco-2 cell adherence of *S.* Agona strains. The adherence assay was conducted in triplicate and are represented as means ± SD. Means of the BIMs were compared to that of the WT. Different letters indicate significance below α = 0.05.

Strain	Total Cells Adhered (log CFU ± SD)
WT	6.22 ± 0.23 ^A^
∆87	6.65 ± 0.33 ^A^
∆95	6.28 ± 0.21 ^A^
∆96	6.01 ± 0.19 ^A^
∆99	6.48 ± 0.38 ^A^
∆99	6.56 ±0.44 ^A^

## Supplementary Materials

Supplementary materials can be found at https://www.mdpi.com/1422-0067/21/5/1883/s1.
