# Supplementary material for: Bacteriophage-Insensitive Mutants of Antimicrobial-Resistant Salmonella Enterica are Altered in their Tetracycline Resistance and Virulence in Caco-2 Intestinal Cells
Source: Int J Mol Sci. 2020 Mar 10;21(5):1883. doi: 10.3390/ijms21051883 (PMC7084636; doi:10.3390/ijms21051883)
Supplement: Supplementary file 1 [file ijms-21-01883-s001.pdf]

SUPPLEMENTARY MATERIALS

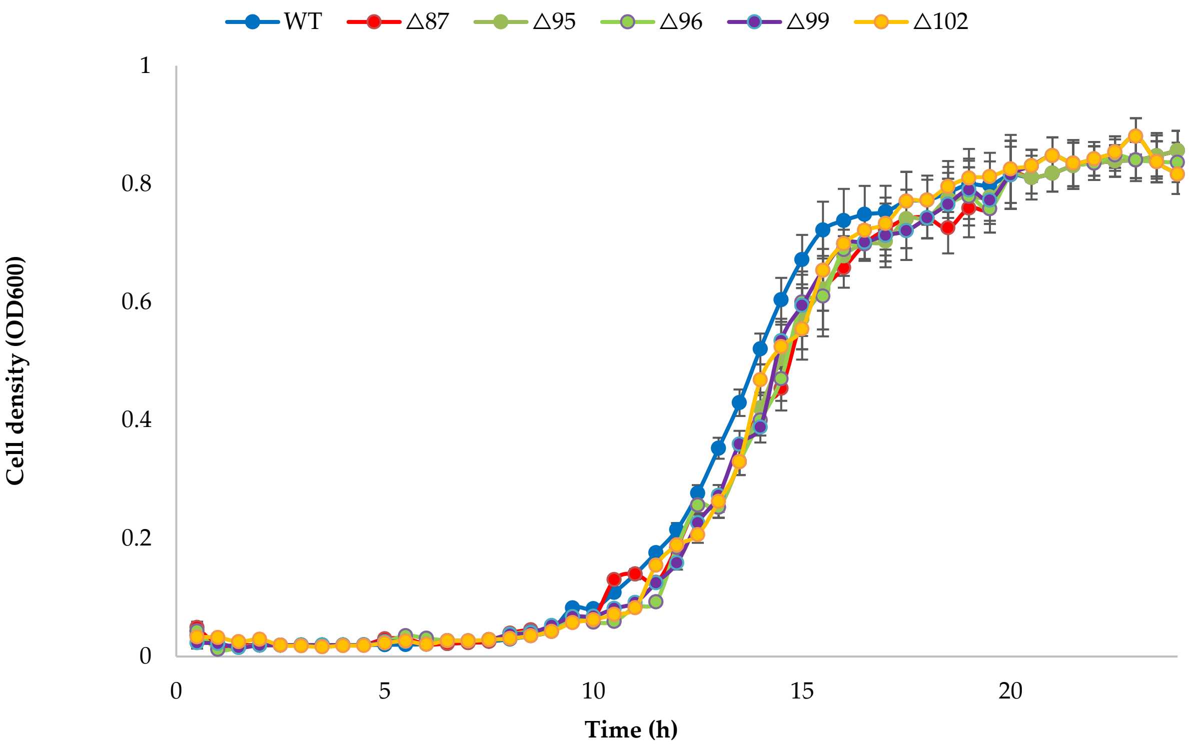

Figure S1. Relative growth rates of the parental and BIM isolates of S. Agona FSL S5-517. Data shown are the means of three biological replicates ± S

International Journal of *Molecular Sciences*

MDPI

Table S1. Mutated genes identified in *S*. Agona BIM Δ87. Mutations shown possess a quality score of >20 and a mapping coverage of >8.

Scaffold	Gene	Strand	Protein/function	Type[a]	Sequence	Alternative sequence[b]	nt position[c]	Impact on polypeptide
1.1	*ccmA*	-	ATP-binding export protein	SNP	A	A>G	609	None
1.1	*ccmC*	-	Heme exporter protein	SNP	C	C>T	240	None
1.1	*ccmC*	-	Heme exporter protein	MNP	TGTCATTCATTAC	CGTAATGCAGTAA	521-531	None
1.1	Non-coding region	N/A	Non-coding	Insertion	TAAAAAA	TGCGAAA	611308	N/A
1.1	Non-coding region	N/A	Non-coding	SNP	T	T>G	661695	N/A
2.1	Non-coding region	N/A	Non-coding	SNP	A	A>T	479242	N/A
3.1	16S rRNA	+	Ribosomal structure	SNP	T	T>C	468	None
3.1	*orfA*	-	Transposase	Insertion	TCCCGCTGACACCCGTCAG	TCCCGCTGACACCCGTTATGGC ATTCAG	245	Frameshift
4.1	Non-coding region	N/A	Non-coding	SNP	G	G>T	44	N/A
4.1	Non-coding region	N/A	Non-coding	SNP	T	T>A	66	N/A
5.1	*ccmH_1*	-	Cytochrome biogenesis protein	SNP	A	A>C	446	None
6.1	*astA*	-	Arginine N-succinyltransferase	SNP	C	C>T	736	His → Tyr
6.1	Non-coding region	N/A	Non-coding	MNP	TT	TT>AC	111166	N/A
6.1	Non-coding	N/A	Non-coding	SNP	T	T>A	111173	N/A

	region							
6.1	Non-coding region	N/A	Non-coding	SNP	C	C>A	111174	N/A
6.1	Non-coding region	N/A	Non-coding	SNP	A	A>C	111176	N/A
6.1	Non-coding region	N/A	Non-coding	SNP	T	T>A	113528	N/A
6.1	Non-coding region	N/A	Non-coding	Insertion	GTGAATGAAGGAT	GTGTAATAACGGAT	113529	N/A
6.1	Non-coding region	N/A	Non-coding	Insertion	ACTTA	AATATCTTATTTGCAGTGGTGAC CCA	113545	N/A
6.1	*solA*	+	N-methyl-L-tryptophan oxidase	SNP	T	T>G	543	None
6.1	*sufD*	-	FeS cluster assembly protein	SNP	G	G>C	846	Trp → Cys
7.1	*argS*	-	Arginine -tRNA ligase	SNP	G	G>T	1034	Arg → Leu
7.1	*cmoB*	-	tRNA U34 carboxymethyltransferase	SNP	T	T>A	241	Ser → Thr
7.1	*narJ*	+	Nitrate reductase molybdenum cofactor assembly chaperone	SNP	G	G>C	424	Val → Leu
7.1	*vgrG1_2*	+	Tip protein/secreted effector; involved in Type VI secretion	Insertion	TT	TCAAGGAT	792	Frameshift
7.1	*vgrG1_2*	+	Tip protein/secreted effector; involved in Type VI secretion	MNP	ATTTT	ATTTT>CGGCC	798	Leu → Phe<br>Phe → Gly<br>Tyr → His
8.1	*IS256*	-	Transposase	SNP	G	G>A	75	None
8.1	Non-coding region	N/A	Non-coding	SNP	T	T>G	50489	N/A
12.1	*purL*	-	Phophoribosylformylglycinamidine	SNP	C	C>A	1428	Asp → Glu

			synthase					
26.1	*impA*	-	Involved in Type IV secretion	Insertion	CTCCC	CTCCGTAGC	767	Frameshift
28.1	Non-coding region	N/A	Non-coding	SNP	A	A>T	26	N/A
29.1	Non-coding region	N/A	Non-coding	SNP	A	A>G	2251	N/A
30.1	23S rRNA	+	Ribosomal structure	Insertion	TG	TCG	505	Frameshift
30.1	23S rRNA	+	Ribosomal structure	SNP	TG	TG>TA	506	None
30.1	Non-coding region	N/A	Non-coding	MNP	CGGCG	CGGCG>CCGAT	1040	N/A
30.1	Non-coding region	N/A	Non-coding	Insertion	AGCGAACGGG	AGCCAGAACGGG	1045	N/A
30.1	Non-coding region	N/A	Non-coding	Insertion	TGT	TCGT	2266	N/A
40.1	16S rRNA	-	Ribosomal structure	MNP	GGAAGTTTT	GGAAGTTTT>AGAACTTTC	1287-1296	None

[a] SNPs clustered in close proximity (i.e., MNPs) are shown on a single line for clarity.
[b] In substitutions, alternate sequences are preceded by the original sequence and separated with a “>” symbol.
[c] Positions are shown for mutations occurring in genes. Positions in the scaffold are shown for mutations occurring in non-coding regions.

Table S2. Mutated genes identified in *S*. Agona BIM Δ95. Mutations shown possess a quality score of >20 and a mapping coverage of >8.

Scaffold	Gene	Strand	Protein/function	Type[a]	Sequence	Alternative sequence[b]	nt position[c]	Impact on polypeptide
1.1	*ccmC*	-	Heme exporter protein	SNP	C	C>T	246	None
1.1	*ccmC*	-	Heme exporter protein	SNP	G	G>C	278	Ala → Pro
1.1	*ccmC*	-	Heme exporter protein	SNP	G	G>A	357	None
1.1	*ccmC*	-	Heme exporter protein	MNP	TGTCATTCATTAC	TGTCATTCATTAC>GGTTATCCACTAT	522-534	None
1.1	*ccmC*	-	Heme exporter protein	SNP	T	T>C	540	None
1.1	*ccmC*	-	Heme exporter protein	SNP	G	G>A	569	None
1.1	*ccmC*	-	Heme exporter protein	SNP	G	G>A	574	Arg → Gln
1.1	*ccmC*	-	Heme exporter protein	SNP	C	C>T	588	None
1.1	*ccmD*	-	Heme exporter protein	SNP	G	G>A	122	Arg → Gln
1.1	*ccmD*	-	Heme exporter protein	SNP	C	C>G	168	None
1.1	*ccmH_2*	-	Cytochrome biogenesis protein	SNP	G	G>A	132	None
1.1	Non-coding region	N/A	N/A	SNP	A	A>C	661695	N/A
1.1	*rfaL*	+	O-antigen ligase	Insertion	GA	GGATA	425	Insertion
1.1	*rfaL*	+	O-antigen ligase	SNP	G	G>A	429	None
1.1	*rfaL*	+	O-antigen ligase	Insertion	TA	TGGGA	442	Insertion
1.1	*rfaY*	-	Lipopolysaccharide core heptose (II) kinase	Insertion	AA	AGCGAAGCCCTAAACTTGTTAAAAA	514	Frameshift
1.1	*rfaY*	-	Lipopolysaccharide core heptose (II) kinase	SNP	T	T>G	538	Asp → Lys

1.1	*rfaY*	-	Lipopolysaccharide core heptose (II) kinase	Insertion	T	TCAAGGA	792	Frameshift
3.1	23S rRNA	+	Ribosome structure	Insertion	AAGCGTGTAG	AGGTAGCGTGCAATAG	1134	Frameshift
3.1	23S rRNA	+	Ribosome structure	Insertion	GACT	GAAGACAACAATTTTCACT	1029	Frameshift
3.1	*orfA*	-	Transposase/transposition	Insertion	TG	TGCAAAGAATG	246	Frameshift
4.1	Non-coding region	N/A	N/A	SNP	G	G>T	44	N/A
4.1	Non-coding region	N/A	N/A	SNP	T	T>A	66	N/A
6.1	*astA*	-	Arginine N-succinyltransferase	SNP	C	C>T	736	His → Tyr
6.1	*solA*	+	N-methyl-L-tryptophan oxidase	SNP	T	T>G	543	None
6.1	*sufD*	-	FeS cluster assembly protein	SNP	G	G>C	846	Trp → Cys
7.1	*argS*	-	Arginine -tRNA ligase	SNP	G	G>T	1034	Arg → Leu
7.1	*cmoB*	-	tRNA U34 carboxymethyltransferase	SNP	T	T>A	241	Ser → Thr
7.1	*narJ*	+	Nitrate reductase molybdenum cofactor assembly chaperone	SNP	G	G>C	424	Val → Leu
7.1	*vgrG1_2*	+	Tip protein/secreted effector; involved in Type VI secretion	Insertion	TT	TCAAGGAT	792	Frameshift
7.1	*vgrG1_2*	+	Tip protein/secreted effector; involved in Type VI secretion	MNP	ATTTT	ATTTT>CGGCC	798	Leu → Phe<br>Phe → Gly<br>Tyr → His
7.1	*vgrG1_2*	+	Tip protein/secreted effector; involved in Type VI secretion	Insertion	AT	AGAACT	810	Frameshift
7.1	*vgrG1_2*	+	Tip protein/secreted effector; involved	Insertion	TC	TCAC	816	Frameshift

			in Type VI secretion					
7.1	*vgrG1_2*	+	Tip protein/secreted effector; involved in Type VI secretion	SNP	G	G>C	820	Gly → Arg
7.1	*vgrG1_2*	+	Tip protein/secreted effector; involved in Type VI secretion	SNP	G	G>T	823	Gly → Stop codon
7.1	*vgrG1_2*	+	Tip protein/secreted effector; involved in Type VI secretion	SNP	A	A>G	825	None
7.1	*vgrG1_2*	+	Tip protein/secreted effector; involved in Type VI secretion	Deletion	AGGA	A---	828	Deletion
7.1	*vgrG1_2*	+	Tip protein/secreted effector; involved in Type VI secretion	SNP	C	C>G	831	Asp → Glu
7.1	*vgrG1_2*	+	Tip protein/secreted effector; involved in Type VI secretion	SNP	C	C>A	836	Ala → Glu
8.1	*mukB*	+	Chromosome partition protein	MNP	CAAA	CAAA>GAAG	1100	Ala → Gly<br>Asn → Ser
12.1	*purL*	-	Phophoribosylformylglycinamidine synthase	SNP	C	C>A	1428	Asp → Glu
26.1	*impA*	-	Involved in Type IV secretion	Insertion	CTCCC	CTCCGTAGC	767	Frameshift
28.1	Non-coding region	N/A	N/A	SNP	A	A>T	26	N/A
30.1	23S rRNA	+	Ribosome structure	Insertion	TG	TCG	505	Frameshift
30.1	Non-coding region	N/A	N/A	Insertion	GCGGCGAGC	GCGGCGATAGTAGC	1039	N/A
34.1	16S rRNA	+	Ribosome structure	Insertion	TC	TAC	1477	Frameshift

[a] SNPs clustered in close proximity (i.e., MNPs) are shown on a single line for clarity.
[b] In substitutions, alternate sequences are preceded by the original sequence and separated with a ">" symbol.
[c] Positions are shown for mutations occurring in genes. Positions in the scaffold are shown for mutations occurring in non-coding regions.

Table S3. Mutated genes identified in *S*. Agona BIM Δ96. Mutations shown possess a quality score of >20 and a mapping coverage of >8.

Scaffold	Gene	Strand	Protein/function	Type[a]	Sequence	Alternative sequence[b]	nt position[c]	Impact on polypeptide
1.1	*ccmA*	-	ATP-binding export protein	SNP	A	A>G	609	None
1.1	*ccmC*	-	Heme exporter protein	SNP	C	C>T	246	None
1.1	*ccmC*		Heme exporter protein	SNP	G	G>C	278	Ala → Pro
1.1	*ccmC*	-	Heme exporter protein	SNP	G	G>A	357	None
1.1	*ccmC*	-	Heme exporter protein	MNP	TGTCATTCATTAC	TGTCATTCATTAC>GGTTATCCACTAT	522-534	None
1.1	*ccmC*	-	Heme exporter protein	SNP	C	C>T	588	None
1.1	*ccmC*	-	Heme exporter protein	SNP	G	G>A	574	Arg → Gln
1.1	*ccmC*	-	Heme exporter protein	SNP	G	G>A	569	None
1.1	*ccmD*	-	Heme exporter protein	SNP	G	G>A	122	Arg → Gln
1.1	*ccmE*	-	Cytochrome biogenesis protein	MNP	TTAC	TTAC>CTAT	24	Arg → Trp
1.1	Non-coding region	N/A	N/A	SNP	A	A>C	661695	N/A
1.1	Non-coding region	N/A	N/A	SNP	A	A>C	611313	N/A
1.1	*rfaL*	+	O-antigen ligase	Insertion	GA	GGATA	425	Insertion
1.1	*rfaL*	+	O-antigen ligase	SNP	G	G>A	429	None

1.1	*rfaL*	+	O-antigen ligase	Insertion	TA	TGGGA	442	Insertion
1.1	*rfaY*	-	Lipopolysaccharide core heptose (II) kinase	Insertion	AA	AGCGAAGCCCTAAACTTGTTAAAAA	514	Frameshift
1.1	*rfaY*	-	Lipopolysaccharide core heptose (II) kinase	SNP	T	T>G	538	Asp → Lys
1.1	*rfaY*	-	Lipopolysaccharide core heptose (II) kinase	Insertion	T	TCAAGGA	792	Frameshift
3.1	*orfA*	-	Transposase/transposition	Insertion	TG	TGCAAAGAATG	246	Frameshift
4.1	Non-coding region	N/A	Non-coding	SNP	G	G>T	44	N/A
4.1	Non-coding region	N/A	Non-coding	SNP	T	T>A	66	N/A
4.1	Non-coding region	N/A	Non-coding	SNP	C	C>G	334561	N/A
5.1	*ccmH_1*	-	Cytochrome biogenesis protein	SNP	A	A>C	446	None
6.1	*astA*	-	Arginine N-succinyltransferase	SNP	C	C>T	736	His → Tyr
6.1	*solA*	+	N-methyl-L-tryptophan oxidase	SNP	T	T>G	543	None
6.1	*sufD*	-	FeS cluster assembly protein	SNP	G	G>C	846	Trp → Cys
7.1	*argS*	-	Arginine -tRNA ligase	SNP	G	G>T	1034	Arg → Leu
7.1	*cmoB*	-	tRNA U34 carboxymethyltransferase	SNP	T	T>A	241	Ser → Thr
7.1	*narJ*	+	Nitrate reductase molybdenum cofactor assembly chaperone	SNP	G	G>C	424	Val → Leu
7.1	*vgrG1_2*	+	Tip protein/secreted effector; involved in Type VI secretion	Insertion	TT	TCAAGGAT	792	Frameshift

7.1	*vgrG1_2*	+	Tip protein/secreted effector; involved in Type VI secretion	MNP	ATTTT	ATTTT>CGGCC	798	Leu → Phe<br>Phe → Gly<br>Tyr → His
7.1	*vgrG1_2*	+	Tip protein/secreted effector; involved in Type VI secretion	Insertion	AT	AGAACT	810	Frameshift
7.1	*vgrG1_2*	+	Tip protein/secreted effector; involved in Type VI secretion	Insertion	TC	TCAC	816	Frameshift
7.1	*vgrG1_2*	+	Tip protein/secreted effector; involved in Type VI secretion	SNP	G	G>C	820	Gly → Arg
7.1	*vgrG1_2*	+	Tip protein/secreted effector; involved in Type VI secretion	SNP	G	G>T	823	Gly →Stop codon
7.1	*vgrG1_2*	+	Tip protein/secreted effector; involved in Type VI secretion	SNP	A	A>G	825	None
7.1	*vgrG1_2*	+	Tip protein/secreted effector; involved in Type VI secretion	Deletion	AGGA	A---	828	Frameshift
7.1	*vgrG1_2*	+	Tip protein/secreted effector; involved in Type VI secretion	SNP	C	C>G	831	Asp → Glu
7.1	*vgrG1_2*	+	Tip protein/secreted effector; involved in Type VI secretion	SNP	C	C>A	836	Ala → Glu
12.1	*purL*	-	Phophoribosylformylglycinamidine synthase	SNP	C	C>A	1428	Asp → Glu
26.1	*impA*	-	Involved in Type IV secretion	Insertion	CTCCC	CTCCGTAGC	767	Frameshift
28.1	Non-coding region	N/A	N/A	SNP	A	A>T	26	N/A

29.1	Non-coding region	N/A	N/A	Insertion	CAAC	TATTACGTTTTGGCTGGTCAAC	3	N/A
29.1	Non-coding region	N/A	N/A	Insertion	GTAC	GTACAACCGAGGTGACTCGTGGCCGTGATGG	2248	N/A
30.1	Non-coding region	N/A	N/A	Insertion	GCGGCGAGC	GCGGCGATAGTAGC	1039	N/A
34.1	16S rRNA	+	Ribosome structure	Insertion	TC	TAC	1477	Frameshift
36.1	Non-coding region	N/A	N/A	Insertion	TGAG	TGAGATTCCCCCAGTA	742	Frameshift

[a] SNPs clustered in close proximity (i.e., MNPs) are shown on a single line for clarity.
[b] In substitutions, alternate sequences are preceded by the original sequence and separated with a ">" symbol.
[c] Positions are shown for mutations occurring in genes. Positions in the scaffold are shown for mutations occurring in non-coding regions.

Table S4. Mutated genes identified in *S*. Agona BIM Δ99. Mutations shown possess a quality score of >20 and a mapping coverage of >8.

Scaffold	Gene	Strand	Protein/function	Type[a]	Sequence	Alternative sequence[b]	nt position[c]	Impact on polypeptide
1.1	*ccmC*	-	Heme exporter protein	SNP	C	C>T	246	None
1.1	*ccmC*	-	Heme exporter protein	SNP	G	G>C	278	Ser → Thr
1.1	*ccmC*	-	Heme exporter protein	SNP	G	G>A	357	None
1.1	*ccmC*	-	Heme exporter protein	SNP	C	C>T	534	None
1.1	*ccmC*	-	Heme exporter protein	SNP	T	T>C	540	None

1.1	*ccmC*	-	Heme exporter protein	SNP	G	G>A	570	Ser → Tyr
1.1	*ccmC*	-	Heme exporter protein	SNP	G	G>A	575	Arg → Leu
1.1	*ccmC*	-	Heme exporter protein	SNP	C	C>T	588	None
1.1	*ccmD*	-	Heme exporter protein	SNP	G	G>A	122	Arg → Gln
1.1	*ccmE*	-	Cytochrome C maturation protein	SNP	C	C>T	228	None
1.1	*ccmE*	-	Cytochrome C maturation protein	SNP	T	T>C	249	None
1.1	*ccmE*	-	Cytochrome C maturation protein	SNP	G	G>T	268	Ala → Ser
1.1	Non-coding region	N/A	N/A	Deletion	GCCCC	G---C	611298	Deletion
1.1	Non-coding region	N/A	N/A	SNP	A	A>C	611314	N/A
1.1	Non-coding region	N/A	N/A	Insertion	TAGAGAAAC	TAGCCACGTAAC	612552	N/A
1.1	Non-coding region	N/A	N/A	Insertion	GGGAGTTCA	GGATAATCTA	612566	N/A
1.1	Non-coding region	N/A	N/A	SNP	T	T>G	661695	N/A
3.1	*orfA*	-	Transposase/transposition	Insertion	TG	TGCAAAGAATG	246	Frameshift
4.1	*ycjG*	+	L-Ala-D/L-Glu epimerase	SNP	A	A>T	296	Gln → Leu
6.1	*astA*	(-)	Arginine N-succinyltransferase	SNP	C	C>T	736	His → Tyr
6.1	*chbC*	-	Selective sugar transport	SNP	G	G>A	275	Gly → Asp
6.1	Non-coding region	N/A	N/A	SNP	T	T>C	111167	N/A
6.1	*solA*	(+)	N-methyl-L-tryptophan oxidase	SNP	T	T>G	543	None
6.1	*sufD*	(-)	FeS cluster assembly protein	SNP	G	G>C	846	Trp → Cys
7.1	*argS*	-	Arginine -tRNA ligase	SNP	G	G>T	1034	Arg → Leu
7.1	*cmoB*	-	tRNA U34 carboxymethyltransferase	SNP	T	T>A	241	Ser → Thr
7.1	*narJ*	+	Nitrate reductase molybdenum cofactor assembly chaperone	SNP	G	G>C	424	Val → Leu

7.1	Non-coding region	N/A	N/A	SNP	C	C>A	111169	N/A
7.1	*vgrG1_2*	+	Tip protein/secreted effector; involved in Type VI secretion	Insertion	TT	TCAAGGAT	792	Frameshift
7.1	*vgrG1_2*	(+)	Tip protein/secreted effector; involved in Type VI secretion	MNP	ATTTT	ATTTT>CGGCC	798	Leu → Phe<br>Phe → Gly<br>Tyr → His
8.1	*mukB*	+	Chromosome partition protein	MNP	CAAA	CAAA>GAAG	1100	Ala → Gly<br>Asn → Ser
8.1	*nfsA*		Oxygen-insensitive NADPH nitroreductase	SNP	A	A>G	251	Asn--> Ser
8.1	Non-coding region	N/A	N/A	SNP	T	T>C	111170	N/A
8.1	*ybjI*	-	5-amino-6-(5-phospho-D-ribitylamino) uracil phosphatase	Deletion	GCGG	G--G	718	Frameshift
9.1	Non-coding region	N/A	N/A	SNP	T	T>C	111172	N/A
10.1	Non-coding region	N/A	N/A	SNP	T	T>G	111173	N/A
11.1	Non-coding region	N/A	N/A	SNP	T	T>C	111175	N/A
12.1	Non-coding region	N/A	N/A	SNP	A	A>G	111176	N/A
13.1	Non-coding region	N/A	N/A	SNP	C	C>T	111177	N/A
30.1	23S rRNA	+	Ribosome structure	MNP	TCT	TCT>TAG	842	None
34.1	16S rRNA	+	Ribosome structure	Insertion	TC	TAC	729	Frameshift

[a] SNPs clustered in close proximity (i.e., MNPs) are shown on a single line for clarity.
[b] In substitutions, alternate sequences are preceded by the original sequence and separated with a ">" symbol.
[c] Positions are shown for mutations occurring in genes. Positions in the scaffold are shown for mutations occurring in non-coding regions.

Table S5. Mutated genes identified in *S.* Agona BIM Δ102. Mutations shown possess a quality score of >20 and a mapping coverage of >8.

Scaffold	Gene	Strand	Protein/function	Type[a]	Sequence	Alternative sequence[b]	nt position[c]	Impact on polypeptide
1.1	*ccmA*	-	ATP-binding export protein	SNP	A	A>G	609	None
1.1	*ccmC*	-	Heme exporter protein	SNP	C	C>T	246	None
1.1	*ccmC*	-	Heme exporter protein	SNP	G	G>C	278	Ser → Thr
1.1	*ccmC*	-	Heme exporter protein	SNP	G	G>A	357	None
1.1	*ccmC*	-	Heme exporter protein	SNP	C	C>T	534	None
1.1	*ccmC*	-	Heme exporter protein	SNP	T	T>C	540	None
1.1	*ccmC*	-	Heme exporter protein	SNP	G	G>A	570	Ser → Tyr
1.1	*ccmC*	-	Heme exporter protein	SNP	G	G>A	575	Arg → Leu
1.1	*ccmC*	-	Heme exporter protein	SNP	C	C>T	588	None
1.1	Non-coding region	N/A	N/A	Insertion	T	G	661695	N/A
1.1	Non-coding region	N/A	N/A	Insertion	GCTACG	GCGGCCTATCTCG	612534	N/A
3.1	23S rRNA	+	Ribosome structure	Insertion	GACT	GAAGACAACAATTTTC ACT	1029	Frameshift
3.1	Non-coding region	N/A	N/A	Deletion	TCAGATTCAGAGTCCG	TCA----------CCG	2296	Frameshift
4.1	Non-coding region	N/A	Non-coding	SNP	G	G>T	44	N/A
4.1	Non-coding region	N/A	Non-coding	SNP	T	T>A	66	N/A
4.1	*rsxC_1*	+	Electron transport complex subunit	SNP	A	A>G	1911	Thr → Ala
5.1	*ccmH_1*	-	Cytochrome biogenesis protein	SNP	A	A>C	447	None

6.1	*astA*	-	Arginine N-succinyltransferase	SNP	C	C>T	736	His → Tyr
6.1	Non-coding region	N/A	Non-coding	MNP	TT	TT>AC	111166	N/A
6.1	*sufD*	-	FeS cluster assembly protein	SNP	G	G>C	846	Trp → Cys
7.1	*argS*	-	Arginine -tRNA ligase	SNP	G	G>T	1034	Arg → Leu
7.1	*cmoB*	-	tRNA U34 carboxymethyltransferase	SNP	T	T>A	241	Ser → Thr
7.1	*narJ*	+	Nitrate reductase molybdenum cofactor assembly chaperone	SNP	G	G>C	424	Val → Leu
7.1	*vgrG1_2*	+	Tip protein/secreted effector; involved in Type VI secretion	Insertion	TT	TCAAGGAT	792	Frameshift
7.1	*vgrG1_2*	+	Tip protein/secreted effector; involved in Type VI secretion	MNP	ATTTT	ATTTT>CGGCC	798	Leu → Phe<br>Phe → Gly<br>Tyr → His
12.1	*purL*	-	Phophoribosylformylglycinamidine synthase	SNP	C	C>A	1428	Asp → Glu
26.1	*icmF*	-	Type VI secretion	SNP	T	T>G	87	None
26.1	*icmF*	-	Type VI secretion	SNP	G	G>A	78	None
28.1	Non-coding region	N/A	N/A	SNP	A	A>T	26	N/A
29.1	Non-coding region	N/A	Non-coding	SNP	A	A>G	2251	N/A
30.1	Non-coding region	N/A	N/A	Insertion	GCGGCGAGC	GCGGCGATAGTAGC	1039	N/A
30.1	Non-coding region	N/A	N/A	SNP	G	G>C	1052	N/A

34.1	16S rRNA	+	Ribosome structure	Insertion	TC	TAC	729	Frameshift

[a] SNPs clustered in close proximity (i.e., MNPs) are shown on a single line for clarity.
[b] In substitutions, alternate sequences are preceded by the original sequence and separated with a “>” symbol.
[c] Positions are shown for mutations occurring in genes. Positions in the scaffold are shown for mutations occurring in non-coding regions.

Table S6. CRISPR elements identified in scaffold 2.1 of the parent and BIM strains of *S*. Agona FSL S5-517.

CRISPR start (nt)	CRISPR end (nt)	Spacer	Spacer sequence	Direct repeat consensus sequence
191881	193007	1	TTTTCAGCCCTTGCCGACTGCGGAACGCCCCT	CGGTTTATCCCCGCTGGCGCGGGGAACAC
		2	ATGGATGGCTCATATCCGGTTTCTGTTTCTGC	
		3	GCGAAATAGTGGGGAAAAACCCCTGGTTAACC	
		4	TAGGCCTTGATACCATCGCTCGCACCTCGTCA	
		5	ATATTGATACTGTTCTCAAGCGCCGTTTTAGC	
		6	TGGCGCCAGAAATATTCATGATCATCGGGATT	
		7	TTCATATTCCAGAAAATGCCTGGGTGATGATC	
		8	GTGGTATCTCCTGTCGACCTTGCCGTTAAAAT	
		9	TATAACTGGCGGGTTTTAGTGTCGTTATAAAA	
		10	TCAGGCTTGACGACAGTCTCCAGCCACTCCTG	
		11	GACCGCGCGGGCTATCGTTCAGGACTATTTTT	
		12	TGGAAGATATTGAAAGCGCCCAATCTTCCCAG	
		13	CTCTGTTACACCGTTATGCACAGACCACACAG	
		14	NTTAATGCAAGCGCCATTAACAAGAAAATGAC	

		15	AGCTCCGGCAGGGTCATGTCCCGGCAGGTGCG	
		16	GTTGTGACGAGTTCGTCGTCTTTTTTTTCGCT	
		17	TAGGGAACCTGGCTAAAGTGTCTGAAGGCTAC	
		18	AATTTTTCTGCGGCCAGCGCGGCCAGCGCCTC	
209293	209809	1	CTGCTCAGCGATAACCGGCAGGTTTAGCCGCT	CGGTTTATCCCCGCTGGCGCGGGGAACAC
		2	TTGCCGACCAGATCAATGACAACGCTAACCTT	
		3	CAGATCACAACGGGTGACGTGATTTTTGGAAA	
		4	TCGTTTTTATCTGCATCTCACTCCCCCTTAAC	
		5	GCCTAAGCCGGGATTTGCTTTAGGCCAGTTGG	
		6	AATAAATGGGATGATCCTGTTGTCCGGGATGC	
		7	TCCATCGCTTTTGCCAATCTATCAGACAATTT	
		8	GGCGACTAACGGCGTGCTGACCAGCCAGTCGC	

International Journal of
Molecular Sciences

44
